# Effects of Pre-warming and Co-warming in Preventing Intraoperative Hypothermia

**DOI:** 10.7759/cureus.35132

**Published:** 2023-02-18

**Authors:** Chandra Mohan, Ravi Madhusudhana

**Affiliations:** 1 Anaesthesia, Sri Devaraj Urs Medical College, Kolar, IND

**Keywords:** hypothermia, co-warming, prewarming, warming devices, temperature, intraoperative hypothermia

## Abstract

Background: Hypothermia is a condition characterized by a decreased body temperature. It takes place when the body is exposed to cold weather or water for a longer period of time. Different types of hypothermia include acute hypothermia, exhaustion hypothermia, and chronic hypothermia. Excessive shivering, breathing difficulty, slurred speech, confusion, drowsiness, a weak pulse, and a loss of consciousness are the symptoms related to hypothermia.

Aims: The aim of this study was to see how effective co-warming and pre-warming are at reducing the risk of intraoperative hypothermia.

Materials and methods: A randomized, prospective, comparative clinical study was conducted in a population of 60 participants. Participants were divided into two groups. Participants in group A received pre-warming for 30 minutes at 40°C before transport to the operation theater and also received co-warming before induction of anesthesia. Group B includes those who received co-warming at 40°C from the point of induction of anesthesia.

Results: The mean age (years) of participants in groups A and B was identified as 43.3 ± 11.84 and 45.93 ± 15.87, respectively. The majority of the participants in the study population were males in groups A and B, with 66.67% and 73.33%, respectively. The medians of core temperature and peripheral temperature at the baseline were identified as 36.80 (36.20 to 37.12) and 32.55 (32.38 to 32.72) in group A. Similarly, it was observed as 36 (35.70 to 36.20) and 32 (31.60 to 32.02) in group B. The medians of core temperature and peripheral temperature after the surgery were identified as 34.50 (34.20 to 35) and 32.65 (31.95 to 33) in group A. Similarly, it was identified as 34 (33.80 to 34.25) and 32 (32.10 to 32.25) in group B.

Conclusion: Our study concluded that it is important to prevent hypothermia in patients undergoing surgery under general anesthesia. Pre-operative and intraoperative warming showed a decrease in the rate of fall in core temperature. Hence, both techniques are effective in reducing hypothermia.

## Introduction

Hypothermia is a condition characterized by a decreased body temperature. It takes place when the body is exposed to cold weather or water for a longer period of time. Different types of hypothermia include acute hypothermia, exhaustion hypothermia, and chronic hypothermia [1]. Excessive shivering, breathing difficulty, slurred speech, confusion, drowsiness, a weak pulse, and a loss of consciousness are the symptoms related to hypothermia.

Anesthesia used, intraoperative warming practices, operating room temperature, and IV infusions of fluids or replacement of blood loss are the elements that can cause hypothermic conditions during the surgery [2].

Intraoperative hypothermia is defined as a core temperature of less than 36°C. It is a frequently occurring complication identified during any surgery [3]. The rate of intraoperative hypothermia in distal gastrectomy was identified at 54%, while in gastroenterological surgery and hip fracture fixation, it was 37% and 17%, respectively [4-6]. Shivering, slurred speech, clumsiness, a weak pulse, a lack of coordination, dizziness, redness of the skin, and a loss of consciousness are some of the complications of intraoperative hypothermia [7-9].

Hypothermia starts to develop within the first hour of surgery in 65% of patients, even with active intraoperative warming. Typically, it occurs immediately after the induction of anesthesia and can cause a decrease of 1.6ºC in the core temperature. Of this reduction, 81% is attributed to the core-to-peripheral redistribution of body heat. It is mainly due to the anesthetic-induced vasodilation, and around 46 kcal of heat is redistributed. This can be halted by increasing the peripheral temperature and reducing the core-periphery temperature gradient through pre-warming [10].

Most of the guidelines prefer at least 30 minutes of pre-warming [11]. But it is difficult to apply the recommended 30 minutes or more of pre-warming when there is a pre-operative holding area. Recently, it was reported that pre-warming for less than 30 minutes is effective [11]. A study by Bräuer et al. [11] suggested that ≤30 minutes of regular pre-warming can effectively reduce perioperative hypothermia.

## Materials and methods

This study was conducted in the Department of Anaesthesiology at Sri Devaraj Urs Medical College, Tamaka, Kolar. All 60 patients admitted for elective surgery done under the Department of Anaesthesiology at Sri Devaraj Urs Medical College, Tamaka, Kolar were considered the study population. The current study was a randomized, prospective, comparative clinical study.

Sample size and sampling method

The sample size included 60 patients divided into two groups of 30 patients each. All the eligible subjects were recruited into the study consecutively by the convenient sampling method.

Inclusion criteria

The inclusion criteria included all patients aged 18 to 65 years, with a BMI of 18.5-25 kg/m2, American Society of Anesthesiologists physical status (ASAPS) class 1 and 2, and patients undergoing elective surgery that is expected to last less than two hours under general anesthesia.

Exclusion criteria

The exclusion criteria included febrile patients, patients with co-morbidities like dysautonomia, thyroid disease, uncontrollable diabetes mellitus with autonomic neuropathy, and Cushing's syndrome, patients affected with peripheral vascular disease (PVD) like Raynaud’s syndrome, and hemodynamically unstable patients who require large amounts of fluid resuscitation.

Formula

The sample size was calculated by using the following formula:

 n = 2s_p_^2 ^[z_1-ἀ/2_ + z_1-ẞ_]^2 ^/ µ_d_^2^;

 s_p_^2^ = s_1_^2^ + s_2_^2^ / 2.

Where s_1_^2^ = standard deviation in the first group; s_2_^2^ = standard deviation in the second group; µ_d_^2^ = mean difference between the samples; ἀ = significance level; and 1-ẞ = power.

Ethical considerations

The study was approved by the institutional ethics committee. Participants who were willing to sign the informed consent were included in the study. The benefits and risks involved in the study and the voluntary nature of participation were explained to the participants before obtaining consent. The confidentiality of the study participants was maintained.

Data collection tools

All the relevant parameters were documented in a structured study proforma.

Methodology

A detailed history of the patient was taken. A complete physical examination was done. Routine investigations were conducted.

An 18G intravenous line was secured, and IV fluids were infused at room temperature. Patients were divided randomly into two groups. Group A received pre-warming for 30 minutes at 40°C before shifting to the operation theater and also received co-warming before induction of anesthesia. Group B received co-warming at 40°C from the point of induction of anesthesia.

A skin probe was used to record the baseline peripheral (thumb) temperature. To detect core body temperature, a nasopharyngeal temperature probe was implanted between the tragus and philtrum after induction of anesthesia.

Statistical methods

The core-to-peripheral temperature gradient (at baseline and the end of surgery) was considered a primary outcome variable. The study group (A vs. B) was considered the primary explanatory variable.

Normality distribution was cross-verified by using a statistical test like Shapiro-Wilk or Kolmogorov's test and visual representation like a Q-Q plot and histograms for all quantitative parameters.

For normally distributed quantitative parameters, the mean values were compared between study groups using an independent sample t-test (two groups), and non-normally distributed parameters were compared between study groups using the Mann-Whitney U test. Data were also represented using a clustered bar chart, an error bar chart, and a box plot.

Categorical outcomes were compared between study groups using the chi-square test or Fisher's exact test. If the overall sample size was <20 or if the expected number in any one of the cells was <5, Fisher's exact test was used. P-value < 0.05 was considered to be statistically significant. IBM SPSS (Statistical Package for Social Sciences; IBM Corp., Armonk, NY) was used for statistical analysis.

## Results

A total of 60 participants were included in the final analysis with 30 participants in group A and 30 participants in group B.

Among the study population, the mean age of the participants was 43.3 ± 11.84 years in group A and 45.93 ± 15.87 years in group B. The mean weight of the participants in group A was 61.03 ± 5.01 kg and 60.2 ± 4.6 kg in group B. There was no statistically significant difference in the mean age and weight between the study groups (P > 0.05) (Table 1).

**Table 1:** Comparison of baseline parameters between the study groups (N = 60)

Parameter	Study group (Mean ± SD)		P-value
	Group A (N = 30)	Group B (N = 30)	
Age (in years)	43.3 ± 11.84	45.93 ± 15.87	0.469
Weight (in kg)	61.03 ± 5.01	60.2 ± 4.6	0.505

Among the study population, there were 10 (33.33%) female participants in group A and eight (26.67%) in group B. There were 20 (66.67%) male participants in group A and 22 (73.33%) in group B. There was no statistically significant difference in gender between the study groups (P > 0.05) (Table 2).

**Table 2:** Comparison of gender between the study groups (N = 60)

Gender	Study group		Chi-square	P-value
	Group A (N = 30)	Group B (N = 30)		
Female	10 (33.33%)	8 (26.67%)	0.317	0.573
Male	20 (66.67%)	22 (73.33%)		

Among the study population, the median pulse rate was 87 (82.75 to 96) bpm in group A and 93 (79.50 to 100) bpm in group B. The median systolic blood pressure was 128.50 (120 to 135.25) mm/hg in group A and 124.50 (118 to 130.25) mm/hg in group B, and the median diastolic blood pressure was 81 (77.75 to 89.50) mm/hg in group A and 81.50 (78 to 86.50) mm/hg in group B. The mean arterial pressure was 97.93 ± 9.41 in group A and 95.63 ± 6.92 in group B. There was no statistically significant difference in vital parameters at the pre-operative stage between the study groups (P > 0.05) (Table 3).

**Table 3:** Comparison of vital parameters at the pre-operative stage between the study groups (N = 60)

Parameter	Study group		P-value
	Group A (N = 30)	Group B (N = 30)	
Pulse rate (bpm) (median (IQR))	87 (82.75 to 96)	93 (79.50 to 100)	0.300
Systolic blood pressure (mm/hg) (median (IQR))	128.50 (120 to 135.25)	124.50 (118 to 130.25)	0.118
Diastolic blood pressure (mm/hg) (median (IQR))	81 (77.75 to 89.50)	81.50 (78 to 86.50)	0.813
Mean arterial pressure (mm/hg) (mean ± SD)	97.93 ± 9.41	95.63 ± 6.92	0.285

Among the study population, the median core temperature at baseline was 36.80ºC (36.20 to 37.12) in group A and 36ºC (35.70 to 36.20) in group B, median baseline peripheral temperature was 32.55ºC (32.38 to 32.72) in group A and 32ºC (31.60 to 32.02) in group B, and mean core to peripheral temperature gradient baseline was 4.21 ± 0.69 in group A and 4.14 ± 0.42 in group B. There was a statistically significant difference in median baseline core temperature and baseline peripheral temperature between the study groups (P < 0.05) while it was not statistically significant for the core to peripheral temperature gradient at baseline (P > 005) (Table 4).

**Table 4:** Comparison outcome parameters at baseline between the study groups (N = 60)

Parameter	Study group		P-value
	Group A (N = 30)	Group B (N = 30)	
Core temperature (in °C) (median (IQR))	36.80 (36.20 to 37.12)	36 (35.70 to 36.20)	<0.001
Peripheral temperature (in °C) (median (IQR))	32.55 (32.38 to 32.72)	32 (31.60 to 32.02)	<0.001
Core to peripheral temperature gradient (mean ± SD)	4.21 ± 0.69	4.14 ± 0.42	0.620

Among the study population, the median core temperature at the end of surgery was 34.50ºC (34.20 to 35.00) in group A and 34ºC (33.80 to 34.25) in group B, the median peripheral temperature at the end of surgery was 32.65ºC (31.95 to 33) in group A and 32.10ºC (32.00 to 32.25) in group B, and mean core to peripheral temperature gradient at the end of surgery was 2.07 ± 0.82 in group A and 1.97 ± 0.49 in group B. There was a statistically significant difference in median core temperature and baseline peripheral temperature at the end of surgery between the study groups (P < 0.05) while it was not statistically significant for the core to peripheral temperature gradient at the end of surgery (P > 005) (Table 5).

**Table 5:** Comparison outcome parameters at the end of surgery between the study groups (N = 60)

Parameter	Study group (mean ± SD)		P-value
	Group A (N = 30)	Group B (N = 30)	
Core temperature (in °C) (median (IQR))	34.50 (34.20 to 35)	34 (33.80 to 34.25)	0.001
Peripheral temperature (in °C) (median (IQR))	32.65 (31.95 to 33)	32 (32.10 to 32.25)	0.014
Core to peripheral temperature gradient (mean ± SD)	2.07 ± 0.82	1.97 ± 0.49	0.582

## Discussion

Temperature is considered one of the major parameters to be monitored in anesthesia. Regional anesthesia and general anesthesia impair thermoregulation. There are studies comparing different techniques to prevent intraoperative hypothermia. The present study was carried out to determine the effectiveness of co-warming and pre-warming in reducing intraoperative hypothermia incidence.

A total of 60 participants were enrolled in the study, with 30 belonging to group A and the remaining 30 to group B.

Participants who received pre-warming for 30 minutes at 40°C before shifting to the operation theater and also received co-warming before induction of anesthesia were categorized into group A, whereas those receiving co-warming at 40°C from the point of anesthesia induction were categorized into group B.

In the present study, the mean ages (in years) of the participants in groups A and B were identified as 43.3 ± 11.84 and 45.93 ± 15.87, respectively, while the mean weights (kg) of the participants in groups A and B were 61.03 ± 5.01 and 60.2 ± 4.6, respectively. The mean age was higher in the co-warming group, while the mean weight was higher in the pre-warming group.

In a study by Adriani et al. [12], the mean age was identified as 49.43 ± 13.74 for the pre-warming + intraoperative warming group, while it was 46.67 ± 15.02 in the intraoperative warming alone. In a study conducted by Xiao et al. [13], 53.81 ± 7.26 and 56.50 ± 6.71 years were the mean ages in the pre-warming and co-warming groups, respectively. The studies by Adriani et al. [12] and Xiao et al. [13] showed an increased mean age as compared to our study results.

Yoo et al. [10] conducted a prospective randomized study on 60 patients in which the mean age and weight were identified as 48.04 ± 17.42 years and 63.51 ± 10.89 kg in the pre-warming group. In another study by Lee et al. [14], the mean age and weight of the pre-warming group were observed as 44.4 ± 9.5 years and 60.3 ± 8.5 kg, respectively, which is comparable to the results of our study.

In the current study, the majority of the participants were identified as male in groups A and B, with 66.67% and 73.33%, respectively. Dhara [15] conducted a prospective interventional study on 40 participants, in which the majority of the participants in the pre-warming and co-warming groups were males at 75% and 60%, followed by females at 25% and 40%, respectively.

Lau et al. [16] conducted a randomized control study on 200 participants, 50.5% of whom were female and 49.5% of whom were male. In another observational prospective study by Prabhakar et al. [17], 50% of the participants were male.

In the present study, the medians of pulse rate, systolic blood pressure, and diastolic blood pressure were identified as 87 (82.75 to 96), 128.50 (120 to 135.25), and 81 (77.75 to 89.50) in group A, while they were identified as 93 (79.50 to 100), 124.50 (118 to 130.25), and 81.50 (78 to 86.50) in group B, respectively. The mean arterial pressure was found to be higher in group A (97.93 ± 9.41) as compared to group B (95.63 ± 6.92).

In the current study, the medians of core temperature and peripheral temperature at the baseline were identified as 36.80 (36.20 to 37.12) and 32.55 (32.38 to 32.72) in group A, while they were observed as 36 (35.70 to 36.20) and 32 (31.60 to 32.02) in group B. Similarly, the mean of the core to peripheral temperature was identified as 4.21 ± 0.69 and 4.14 ± 0.42 in groups A and B, respectively.

Shenoy et al. [18] conducted a study on 60 patients in which the medians of core temperature (°C), peripheral temperature (°C), and core to peripheral temperature gradient (°C) at the baseline were identified as 36.1 (0.8), 32.9 (2.4), and 3.4 (1.7, 4.5) in the pre-warming group, while they were identified as 36 (0.58), 32.1 (2.8), and 3.5 (1.8, 5.1), respectively, in the co-warming group, which was an increased median as compared to our study results. Shenoy et al. [18] showed similar results to our study.

Just et al. [19] performed a study on 16 participants in which the mean of core temperature (°C) and peripheral temperature (°C) at the baseline was identified as 36.6 ± 0.1 and 32.3 ± 0.2, respectively, in the pre-warming group.

Similarly, Kim et al. [20] conducted a study on 40 patients in which the mean of core temperature (°C), peripheral temperature (°C), and core to peripheral temperature (°C) at the baseline in the pre-warming group was 36.7 ± 2.4, 30.5 ± 0.2, and 6.2 ± 2.7, respectively.

In the present study, the medians of core temperature and peripheral temperature at the end of the surgery were identified as 34.50 (34.20 to 35) and 32.65 (31.95 to 33) in group A, while they were identified as 34 (33.80 to 34.25) and 32 (32.10 to 32.25) in group B. Similarly, the mean of the core to peripheral temperature was identified as 2.07 ± 0.82 and 1.97 ± 0.49 in groups A and B, respectively.

In Shenoy et al.'s study, the core temperature (°C), peripheral temperature (°C), and core to peripheral temperature gradient (°C) at the end of the surgery were identified as 34.6 (1.2), 33.0 (2.4), and 2.8 (0.9, 6.4), respectively. Whereas it was 34.3 (1.3), 32.1 (2.8), and 2.9 (0.6, 6.2), respectively, which resembles our study results.

In another study by Just et al., the mean of core temperature (°C) and peripheral temperature (°C) at the end of the surgery in the pre-warming group was 36.3 ± 0.1 and 33.3 ± 0.3, respectively.

In Kim et al.'s [20] study, the mean of core temperature (°C), peripheral temperature (°C), and core to peripheral temperature (°C) at the end of the period was identified as 35.6 ± 0.5, 29.1 ± 4.0, and 6.6 ± 4.2, respectively, in the pre-warming group.

## Conclusions

Our study concluded that it is important to prevent hypothermia in patients undergoing surgery under general anesthesia. Post-operative and intraoperative warming showed a decrease in the rate of fall in core temperature. Hence, both techniques are effective in reducing hypothermia.
